# Identification of age-dependent motor and neuropsychological behavioural abnormalities in a mouse model of Mucopolysaccharidosis Type II

**DOI:** 10.1371/journal.pone.0172435

**Published:** 2017-02-16

**Authors:** Hélène F. E. Gleitz, Claire O’Leary, Rebecca J. Holley, Brian W. Bigger

**Affiliations:** Stem Cell & Neurotherapies, Division of Cell Matrix Biology & Regenerative Medicine, Faculty of Biology, Medicine and Health, The University of Manchester, Manchester, United Kingdom; Nathan S Kline Institute, UNITED STATES

## Abstract

Severe mucopolysaccharidosis type II (MPS II) is a progressive lysosomal storage disease caused by mutations in the *IDS* gene, leading to a deficiency in the iduronate-2-sulfatase enzyme that is involved in heparan sulphate and dermatan sulphate catabolism. In constitutive form, MPS II is a multi-system disease characterised by progressive neurocognitive decline, severe skeletal abnormalities and hepatosplenomegaly. Although enzyme replacement therapy has been approved for treatment of peripheral organs, no therapy effectively treats the cognitive symptoms of the disease and novel therapies are in development to remediate this. Therapeutic efficacy and subsequent validation can be assessed using a variety of outcome measures that are translatable to clinical practice, such as behavioural measures. We sought to consolidate current knowledge of the cognitive, skeletal and motor abnormalities present in the MPS II mouse model by performing time course behavioural examinations of working memory, anxiety, activity levels, sociability and coordination and balance, up to 8 months of age. Cognitive decline associated with alterations in spatial working memory is detectable at 8 months of age in MPS II mice using spontaneous alternation, together with an altered response to novel environments and anxiolytic behaviour in the open-field. Coordination and balance on the accelerating rotarod were also significantly worse at 8 months, and may be associated with skeletal changes seen in MPS II mice. We demonstrate that the progressive nature of MPS II disease is also seen in the mouse model, and that cognitive and motor differences are detectable at 8 months of age using spontaneous alternation, the accelerating rotarod and the open-field tests. This study establishes neurological, motor and skeletal measures for use in pre-clinical studies to develop therapeutic approaches in MPS II.

## Introduction

Mucopolysaccharidosis type II (MPS II, OMIM #309900 [1]), also known as Hunter syndrome, is an X-linked lysosomal storage disorder that affects 1.3 per 100,000 male live births [2–4]. MPS II is caused by mutations in the *IDS* gene, leading to a deficiency in iduronate-2-sulfatase enzyme (EC 3.1.6.13), and results in impaired degradation of both heparan sulphate and dermatan sulphate and subsequent unregulated accumulation in the lysosomal compartment [5]. MPS II is often classified as either attenuated or severe, although it is likely more of a continuum between two extremes, depending on the severity of symptoms [4]. MPS II patients present with chronic and progressive multi-system disease affecting a multitude of different organs including the bones, joints and heart. Severe MPS II includes progressive neurocognitive decline, skeletal abnormalities known as dysostosis multiplex, short stature, joint stiffness and hepatosplenomegaly [4, 6]. Death usually occurs in mid-teens due to obstructive airway disease and cardiac failure [5, 7, 8].

Enzyme replacement therapy (ERT) is the only approved treatment for MPS II, and has been used to treat the somatic symptoms in MPS II patients regardless of disease severity [9, 10]. However, the blood-brain barrier prevents replacement enzyme from crossing into the central nervous system (CNS) from the bloodstream and therefore considerably limits therapeutic benefits to MPS II patients that are cognitively affected. ERT is administered chronically by weekly intravenous infusions and this intense therapeutic schedule has been associated with severe anaphylactic reactions to the replacement enzyme [10, 11], which may ultimately decrease the efficacy of the treatment [12]. Haematopoietic stem cell transplantations, although efficacious to treat neurological symptoms in other MPS disorders [13–15], have been particularly variable in treating the neurological symptoms of severe MPS II and are associated with high morbidity and mortality [16, 17].

No approved therapy is currently used to treat the neurological symptoms associated with severe MPS II, although a variety of therapeutic strategies are under development. Gene therapy involving second-generation lentiviral vector-mediated stem cell gene therapy [18] and direct injections of various adeno-associated viral vectors [6, 19, 20] are under investigation to treat the CNS. Animal models of MPS disorders have been extensively used for therapeutic development and to gain a thorough understanding of disease pathology, particularly with the development of ERT [21] and other therapies. The identification of reliable outcome measures in mouse models can often be translated to clinical practice, especially where related to neurological symptoms. For examples, very similar circadian rhythm alterations [22] first identified in the MPS IIIB mouse model have also been detected in MPSIII patients [23]. Hyperactive behaviour, originally noted in observations of patients, has been observed in mouse models of MPS IIIA [24], MPS IIIB [25] and MPS IIIC [26], and similarly confirmed in MPS III patients [27]. These types of cross-species behavioural similarities allow for greater confidence in proof of concept studies in animal models translating to successful outcomes in MPS patients.

The most widely used murine model of MPS II produces a biochemical and pathological phenotype which mimics features of the human disease and provides a good basis for novel therapeutic development [6, 28]. Phenotype onset in the MPS II mouse is usually at 12–16 weeks of age and progressively worsens throughout adult life until death at about 60–70 weeks of age [6]. Skeletal abnormalities can be detected as early as 10 weeks of age, and glycosaminoglycan accumulation in urine is detected by 6 weeks of age. Changes in anxiety-related behaviour and locomotor activity were detected between 36 and 40 weeks of age [6, 29], and differences in spatial working memory were identified in 36-week-old mice although the study involved a small sample size [18]. Short and long term memory were recently evaluated in 4–5 months-old MPS II mice using spontaneous alternation, contextual fear conditioning and novel object recognition, showing significant changes in long-term memory only [20]. Some aspects of cognition and neuropsychological phenotype in the MPS II mouse model have been evaluated at distinct time points, but it remains unclear at which age behavioural analysis may be most appropriate. In particular, there is little data describing the time course of behavioural abnormalities in the MPS II mouse model, therefore choosing the right time point for therapeutic intervention is also difficult. We therefore aim to establish robust and reliable tests to evaluate progressive behavioural phenotypes related to MPS II, allowing validation of novel therapies to treat CNS and skeletal abnormalities in MPS II.

## Materials and methods

### Mouse colony maintenance

Female heterozygous for the X-linked *Ids* allele were kindly obtained from Prof. Joseph Muenzer (University of North Carolina, Chapel Hill, USA) and bred with wild-type (WT) C57BL/6J males (Envigo, Alconbury, UK) to obtain WT males and females, and affected hemizygous males and carrier females. WT littermates were used as controls throughout. Mice were housed in individually ventilated cages with *ad libitum* access to food and water, and were kept in a 12-hour light/dark cycle. Male mice were used in this study, with WT littermates used as controls and housed in groups of 2–5. All procedures were ethically approved by the University of Manchester Ethical Review Process under UK Home Office regulations and project licence PPL 40/3658.

### Genotyping of the MPS II mouse model

The MPS II mouse model was developed by removing exons 4 and part of exon 5 of *Ids* and replacing them with the neomycin resistance gene [6, 28]. Genotyping was performed by PCR amplification using genomic DNA extracted from ear punches using the GenElute Mammalian Genomic DNA Miniprep Kit (Sigma Aldrich) according to the manufacturer’s instructions. *Ids* was amplified by PCR using the forward primer 5’ GGGGAGGAGCTACTTGCATAGTTG 3’ and reverse primers 5’ AGGTGGAAAAGACCAGCTATATGG 3’ and 5’ AAAAGAGGACTGCGTGTGGG 3’. Genotype was determined from DNA fragment pattern, where the WT and mutant (MUT) alleles were 152 and 181 bp in size, respectively.

### Behavioural testing

Independent cohorts of 2-, 4-, 6- or 8-months-old male WT and MPS II mice were analysed for phenotypic differences using a variety of behavioural tests, as described below (2 months, WT n = 10, MPS II n = 10; 4 months WT n = 8–11, MPS II n = 7–9; 6 months, WT n = 8–10, MPS II n = 8–10; 8 months, WT n = 7–10, MPS II n = 8–10, total number = 80). Additional independent cohorts of 8-months-old WT and MPS II mice were used for the elevated-plus maze, sociability, social novelty preference and rotarod tests (WT n = 16, MPS II n = 12). All behavioural tests were performed on consecutive days at the same time points by the same researcher.

#### Open-field

Male WT and MPS II mice were analysed using the open-field test, as previously described [24]. All open-field tests were performed 1.5 hours into the 12-hour light cycle. Mice were placed in the centre of a matt white acrylic open-field arena (Width: 450mm, depth: 450mm, height: 500mm, 127 lux), recorded for 60 minutes. Distance moved, frequency of entry into the centre and duration of time spent exploring the centre were analysed using Ethovision XT11.5 software (Noldus, Wageningen, the Netherlands).

#### Spontaneous alternation

Spatial working memory was assessed in independent cohorts of 2-, 4-, 6- and 8-months-old mice using the spontaneous alternation test [30–32]. Spontaneous alternation was evaluated in a single 10-min trial in a Y-maze consisting of three identical arms (40 x 10 x 20 cm) and each mouse was allowed to see distal spatial landmarks. The test mouse was placed in the middle of the three arms and allowed to explore freely. Arm entry was considered successful when hind paws were placed in the arm in full. Spontaneous alternation was described as successive entries into three arms, in overlapping triplet sets. The effect was calculated as percent alternation = [no. of alternations / (total number of arm entries– 2)] x 100. Total entries were recorded as an indication of ambulatory activity and mice that performed fewer than 12 entries in 10 min were excluded from the analysis (n = 1) [33].

#### Inverted screen test

The inverted screen test was performed as previously described [25]. Briefly, the mouse was placed on a square mesh (470mm x 470mm, 13mm x 13mm squares) and the screen was rotated 180° over 2 seconds. Each mouse was suspended upside down over a padded surface, and the number of rear leg moves and suspension time were recorded for a maximum of 120 seconds.

#### Horizontal bar test

The horizontal bar test was performed as described previously [24, 25]. Briefly, a 300mm metal wire (diameter: 2mm) was secured 320mm above padding between two wooden columns. The mouse gripped the centre of the wire and the time to fall or reach either side column was recorded up to 120 seconds. Crossing the bar was scored as 240 minus the number of seconds taken to cross the bar, remaining on the bar was scored as 120, and falling off the bar was recorded as the number of seconds taken to fall. Three practice runs were allowed followed by a 10 minute rest prior to three recorded trials.

#### Elevated-plus maze

The elevated plus maze paradigm uses the innate aversion of rodents towards open areas and spontaneous exploratory behaviour, and was performed as previously described [30]. Briefly, the plus maze consisted of two closed arms (30 cm x 5 cm) surrounded by clear plastic walls (15 cm) and two open arms (30 cm x 5 cm) raised 40 cm off the floor. 8-months-old male mice were placed in the centre of the maze facing one of the open arms and experiments were recorded over a 5 minute period. Time spent in closed and open arms, frequency of entry into the arms and total distance moved over 5 minutes were analysed using Ethovision XT11.5 software.

#### Sociability and social novelty preference

The social novelty test was performed as previously described [30, 32]. Briefly, a rectangular, three-chambered box with clear dividing walls and 4 x 4cm openings was used. Tested mice were allowed free, unrestricted access to all chambers. Small wire cages were placed in the outermost corners of the left and right chambers. All chambers were cleaned with ethanol and fresh bedding was replenished between each trial. The social novelty test consists of three separate phases. The habituation phase allows the mouse to freely explore all three chambers for 10 minutes. The sociability phase consists of adding an unfamiliar, age- and sex-matched conspecific mouse to one of the small wire cages in either the right or left chamber. The test mouse was allowed to explore all three chambers for 10 minutes. The social novelty preference phase consists of adding a second unfamiliar mouse to the empty wire cage, before allowing the tested mouse to explore all three chambers for a further 10 minutes. Time spent in various chambers was analysed using Ethovision XT11.5 software.

#### Rotarod motor learning

The rotarod test was used to evaluate motor coordination, balance and ataxia as previously described with minor modifications [30]. At 2, 4, 6 and 8 of months of age, independent cohorts of male mice were trained on the rotarod (Ugo Basile, Varese, Italy) across three training trials (4 rpm for 120 seconds; 4 rpm for 300 seconds; 4–40 rpm over 300 seconds) with a 30 minute interval between each session. Three test trials were carried out 24 hours post-training. For test trials, the rotarod rotated at an accelerating speed of 4 to 40 rpm over 300 seconds, with a 5 minute rest between each trial. Latency to fall was recorded for all training and test trials, and latency to fall off was calculated as percentage of total trial time.

### X-ray imaging of WT and MPS II mice

Independent cohorts of 2- (WT n = 8, MPS II n = 7), 4- (WT n = 6, MPS II n = 5), 6- (WT n = 4, MPS II n = 6) and 8-months-old (WT n = 6, MPS II n = 6) WT and MPS II mice were radiographed using an X-ray specimen radiography system (Faxitron) and envelope-sealed Amersham Hyperfilm MP X-ray film (GE Healthcare Life Sciences). Mice were culled by exposure to carbon dioxide gas in a rising concentration and radiographed for 55 seconds at 32 kV. Individual bone lengths and widths were measured from scanned radiographic images and analysed using ImageJ software.

### Statistical analysis

Statistical analysis was performed using JMP software (SAS Institute Inc, Cary, NC, USA). For the comparison of separate WT and MPSII cohorts of different ages in open-field, spontaneous alternation, inverted screen, horizontal bar, social novelty preference and elevated-plus maze tests, two-tailed parametric unpaired t-tests were applied for individual group comparison with significance set at p<0.05. A two-way ANOVA for repeated measures with genotype and time factors, followed by Tukey’s multi-comparison test, was performed for distance moved and time in the centre of the open-field. A two-way ANOVA with genotype and time as independent factors, followed by Tukey’s multi-comparison test, was performed for inverted screen, horizontal bar tests, and x-ray analysis. Significance was set at p<0.05 and is indicated by an asterisk in each figure.

## Results

### Activity and exploration in the open-field

Exploratory behaviour in a novel environment in the MPS II mouse model was tested in an open-field arena. Anxiety and habituation to a novel environment are measured in the first 10 minutes, and hyperactivity is examined over the entire 60 minute testing period.

**Fig 1:**
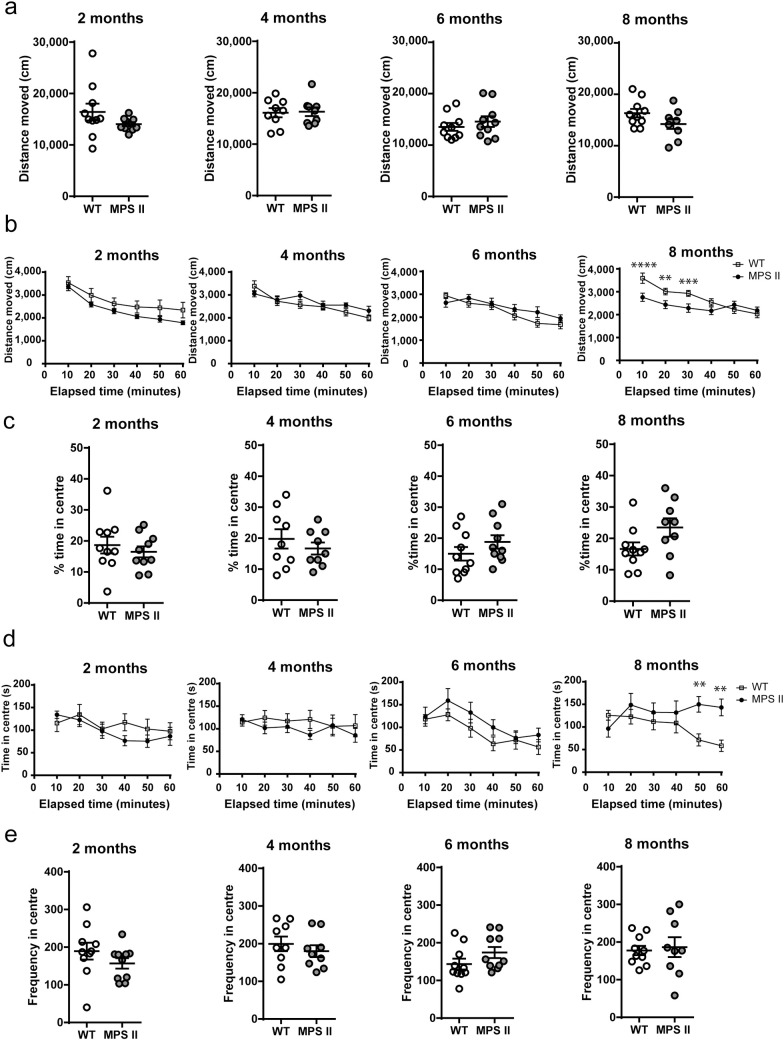
Exploratory activity and anxiety-related behaviour in MPS II mice. Distance moved over 60 minutes (a), distance moved per 10 minute interval (b), percentage of time spent in the centre over 60 minutes (c), percentage time spent in the centre in 10 minute intervals (d) and the frequency of entry into the centre (e) were analysed in independent cohorts of 2- (WT n = 10, MPS II n = 10), 4- (WT n = 9, MPS II n = 9), 6- (WT n = 10, MPS II n = 10) and 8-months-old (WT n = 10, MPS II n = 9) mice. Results are shown as mean ± SEM. ** = p<0.01, *** = p<0.001, **** = p<0.0001, WT vs MPS II.

Independent cohorts of 2-, 4-, 6- and 8-months-old WT or MPS II mice showed no significant differences in the total distance moved over 60 minutes (Fig 1A, S1 Table). Analysis of 10-minute time intervals showed that locomotor activity decreased progressively over the test period in both WT and MPS II mice at 2 months [effect of time, F_5, 45_ = 28.04, p <0.0001], 4 months [F_5, 45_ = 22.65, p<0.0001], 6 months [F_5, 45_ = 26.08, p<0.0001] and 8 months [F_5, 45_ = 20.8, p<0.0001]. In the 8-months-old cohort (Fig 1B, S3 Table), MPS II mice displayed reduced activity in the first 10 minutes of the test compared to WT controls [genotype × time interaction, F_5, 45_ = 10.92, p <0.0001], suggesting a change in habituation to a novel milieu, which is measured in the first 10 minutes.

Additionally, the open field paradigm can be used to assess anxiety-like behaviour in rodents. When mice are introduced to an open field apparatus they are inclined to explore peripheral zones of the apparatus, a phenomenon called thigmotaxis [34]. No significant differences were detected in all cohorts in the time spent exploring the centre zone of the apparatus over the full 60 minutes of the test (Fig 1C, S1 Table). However, exploration of the central zone decreased progressively over the test 10-minute intervals and revealed a time effect at 2 [effect of time, F_5, 45_ = 5.771, p = 0.0003] and 6 months of age [effect of time, F_5, 45_ = 9.47, p<0.0001]. 8-months-old MPS II mice spent more time in the centre in the later stages of the open-field test [40–50min, p = 0.0047, 50–60min, p = 0.0017] compared to WT controls [genotype × time interaction, F_5, 45_ = 5.347, p = 0.0006] (Fig 1D, S2 Table). This decrease in thigmotaxis in the 8-months cohort suggests an anxiolytic phenotype in older MPS II mice. No changes in the frequency of entry into the centre were observed at any time points over the duration of the full 60-minute test (Fig 1E, S1 Table).

### Immediate spatial working memory

**Fig 2:**
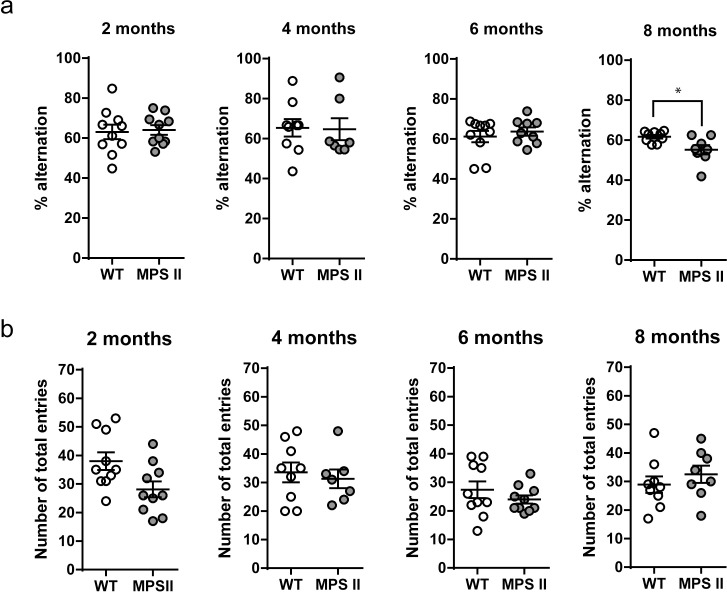
Spatial working memory deficits in MPS II mice. Spontaneous alternation (a) and total number of entries (b) in the Y-maze were analysed as measures of working memory and ambulatory activity in independent cohorts of 2- (WT n = 10, MPS II n = 10), 4- (WT n = 9, MPS II n = 7), 6- (WT n = 10, MPS II n = 10) and 8-months-old (WT n = 9, MPS II n = 8) male mice. Results are shown as mean ± SEM. * = p<0.05 (t-test), WT vs. MPS II.

The spontaneous alternation test using the Y-maze was used to assess spatial working memory in 2-, 4-, 6- and 8-months WT and MPS II mice and investigate the cognitive function of the MPS II mouse model by exploiting their innate preference to explore novel arms compared to recently explored arms of the maze. Interestingly, cognitive dysfunction was not apparent in MPS II mice until 8 months of age, where MPS II mice had a reduction percentage alternation compared to WT controls (Fig 2A, S4 Table)[t_15_ = 2.72, p = 0.0158]. Total number of arm entries was used as a control measure of locomotion; no differences were detected in any cohort (Fig 2B, S4 Table).

### Neuromuscular phenotype

**Fig 3:**
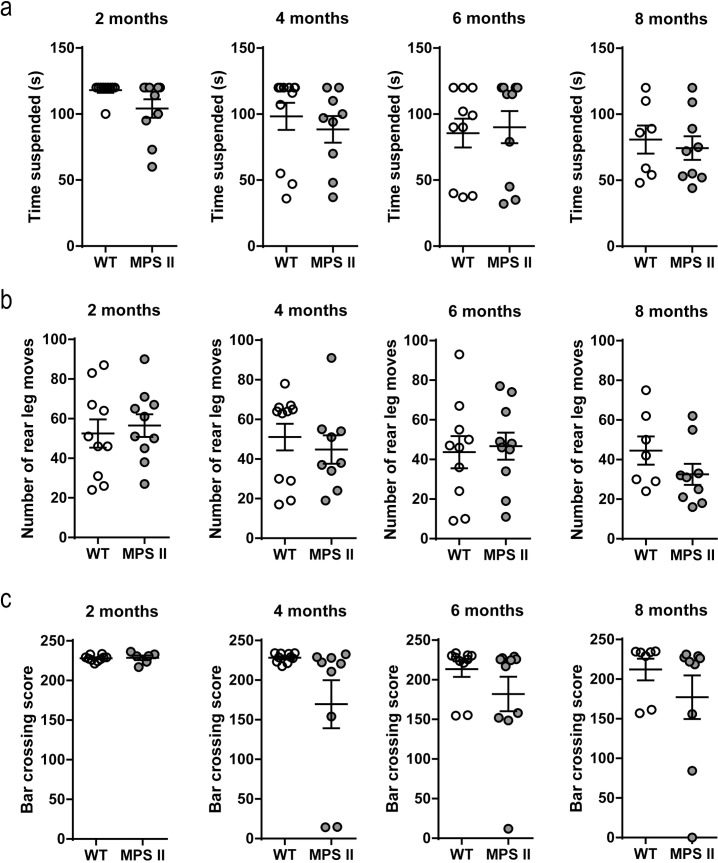
Neuromuscular phenotype in MPS II mice. Time suspended (a) and the number of hind leg moves (b) were monitored using the inverted screen test. The bar crossing score (c) was obtained using the horizontal bar crossing test in independent WT and MPS II cohorts of 2- (WT n = 10, MPS II n = 10), 4- (WT n = 11, MPS II n = 9), 6- (WT n = 10, MPS II n = 10) and 8-months-old (WT n = 7, MPS II n = 9) male mice. Results are shown as mean ± SEM. p<0.05, WT vs. MPS II.

The inverted screen and bar crossing tests were used to measure neuromuscular strength and further understand the skeletal phenotype associated with the MPS II mouse model. When comparing performance of independent cohorts of WT and MPS II mice on the time spent gripping the inverted screen, no significant differences were detected at any age between the two genotypes (Fig 3A, S5 Table). However, time suspended on the inverted screen progressively worsens with age irrespective of genotype [effect of age, F_3, 68_ = 4.206, p = 0.0087]. The number of leg moves using hind limbs showed no differences with age or genotype (Fig 3B, S5 Table). In the bar crossing test, a decrease in neuromuscular strength was observed in MPS II mice overall [effect of genotype, F_1, 68_ = 8.364, p = 0.0051], but no significant age or age × genotype interactions were observed (Fig 3C, S5 Table).

### Ataxia, motor coordination and balance

**Fig 4:**
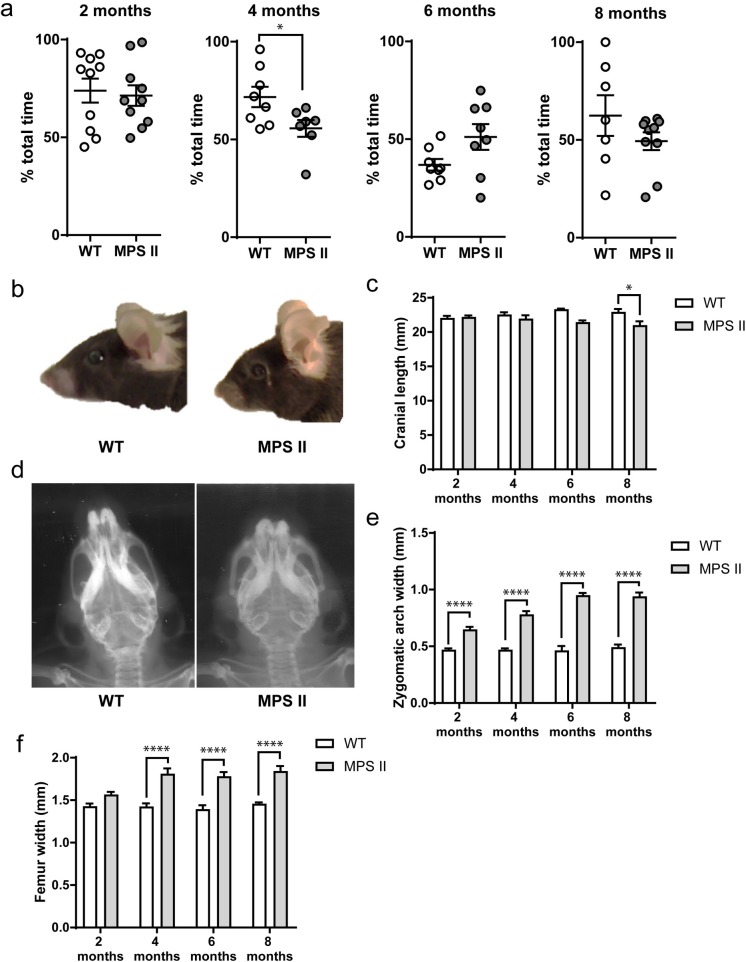
Motor function and skeletal abnormalities in MPS II mice. Percentage of time spent on the accelerating rotarod (a) was measured over three separate trials in independent 2- (WT n = 10, MPS II n = 10), 4- (WT n = 8, MPS II n = 7), 6- (WT n = 8, MPS II n = 8) and 8-months-old (WT n = 7, MPS II n = 10) WT and MPS II cohorts. Results are shown as mean ± SEM. * = p<0.05 (t-test), WT vs. MPS II. Facial abnormalities (b) were seen in photographs of 6.5-months-old MPS II mice. Cranial length (c), zygomatic arch abnormalities (d-e) and femoral width (f) in independent cohorts of 2- (WT n = 8, MPS II n = 7), 4- (WT n = 6, MPS II n = 5), 6- (WT n = 4, MPS II n = 6) and 8-months-old (WT n = 6, MPS II n = 6) WT and MPS II mice were taken using x-ray scans. Results are shown as mean ± SEM. * = p<0.05, **** = p<0.0001, (2-way ANOVA, Tukey’s post-hoc test).

In humans, MPS II features severe skeletal abnormalities in both the attenuated and severe forms, and is generally difficult to correct using currently available therapies. Therefore, models of skeletal abnormalities associated with MPS II should be closely monitored for changes that will impact sensory-motor performance. Motor coordination, balance and ataxia were measured using an accelerating rotarod apparatus in independent cohorts of WT and MPS II mice at various ages. Interestingly, differences in rotarod performances were only detected at 4 months of age (Fig 4A, S6 Table), with MPS II mice performing significantly worse than WT controls [t_13_ = 2.345, p = 0.0355]. This trend was not seen at 6- or 8-months of age due to variability in WT control groups.

X-ray imaging of independent cohorts of 2-, 4-, 6- and 8-months-old mice also showed significant changes in cranial length. This effect was only observed in the 8-months-old cohort [age × genotype interaction, F_3_, _39_ = 3.8, p = 0.0175, effect of genotype, F_1, 39_ = 15.41, p = 0.0003], where 8-months-old MPS II mice have significantly shorter cranium lengths than WT controls (p = 0.016) (Fig 4B and 4C, S7 Table). Additionally, progressive alterations in the width of zygomatic arches of MPS II mice were detected [effect of age, F_3, 39_ = 20.42, p<0.0001, age × genotype interaction, F_3, 39_ = 17.71, p<0.0001]. At all ages examined, zygomatic arches remained significantly different from WT controls [p<0.0001] (Fig 4D and 4E, S7 Table). Furthermore, MPS II mice had significantly thicker femurs than WT littermates (Fig 4F, S7 Table); consistent with zygomatic arch width, this effect exacerbates with age [effect of age, F_3, 39_ = 5.528, p = 0.0029, age × genotype interaction, F_3, 39_ = 4.828, p = 0.0059]. Overall, this data highlights the progressive worsening of skeletal phenotype with age, as often described in MPS II patients.

### Anxiety, sociability, social novelty preference and coordination at 8 months of age

Since most of the changes that we observed were only apparent in 8-months-old mice, an additional independent cohort of 8-months-old WT and MPS II male mice were used to further investigate anxiety-related behaviour using the elevated-plus maze and sociability and social novelty preferences. Additionally, these cohorts were used to further validate the accelerating rotarod as a suitable test to measure coordination and balance at 8 months of age in MPS II, which was previously unclear due to wide variability in the WT control group (Fig 4A, S6 Table).

**Fig 5:**
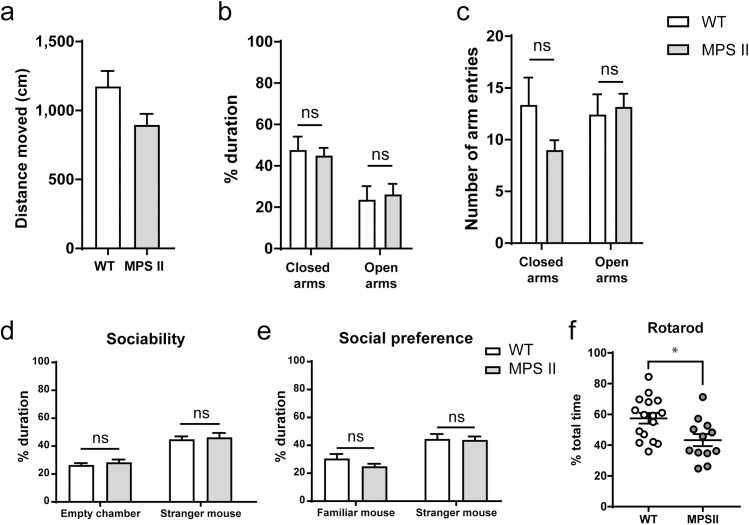
Anxiety-related behaviour, sociability and social novelty preference, and motor coordination in 8-months-old MPS II mice. Distance moved (a), percentage of time spent in closed or open arms (b) and the total number of entries into each arm (c) was measured in the elevated-plus maze in an independent cohort of 8-months-old mice (WT n = 16, MPS II n = 12). Sociability (d) and social preference (e) were measured using a three-chambered testing paradigm and (f) the percentage of time spent on the accelerating rotarod was measured and is shown as the average of 3 trials. Results are shown as mean ± SEM. * = p<0.05, WT vs. MPS II.

The elevated plus maze paradigm was used as an additional measure of anxiety, where rodents innately prefer to explore novel environments but are also reluctant to leave enclosed spaces. The elevated-plus maze is 40 cm above floor level, creating another level of anxiety for the tested mice. No significant differences were detected between the 8-months-old WT and MPS II cohorts (n = 12–16) in the distance moved over the duration of the test (Fig 5A, S8 Table). WT and MPS II mice spent more time in the closed arms, with no significant differences between the two genotypes, suggesting no differences in anxiety-related behaviour (Fig 5B, S8 Table). The number of arm entries into closed and open arms was also monitored as a measure of locomotor activity and showed no differences at 8 months of age (Fig 5C, S8 Table).

Behavioural assays have been described that measure the preference of mice for initiating social interactions with novel conspecifics. Mice, as social animals, prefer to spend more time with other mice and will generally favour novel conspecifics over familiar ones. To this effect, we investigated the sociability status and social novel preference of 8-months-old WT and MPS II mice (n = 12–16). A three-chambered maze was used in a three-part test, where mice are habituated to the maze before being introduced to an age- and sex-matched conspecific mouse for 10 minutes. This sociability phase revealed no significant differences between WT and MPS II mice, as both cohorts spend more time in the chamber containing the novel mouse over the empty chamber [effect of chamber, F_1,52_ = 60.77, p<0.0001] (Fig 5D, S9 Table). In the social novelty preference phase, a second age- and sex-matched conspecific mouse was introduced in the previously empty chamber as the novel intruder, and the test mouse monitored for a further 10 minutes. Test mice now spent more time in the chamber containing the novel stranger mouse than in the opposite chamber containing the (now familiar) mouse [effect of chamber, F_1,52_ = 26.35, p<0.0001]. No significant differences were observed between WT and MPS II groups (Fig 5E, S9 Table).

Additional validation of the accelerating rotarod was also performed using additional cohorts of 8 months-old mice, which were independent of groups used in our time course experiments. MPS II mice spent significantly less time on the rod than WT controls [t_26_ = 2.713, p = 0.0117], which were observed for excessive weight gain, and indicate a significant motor phenotype at 8 months of age (Fig 5F, S6 Table).

## Discussion

The aim of this study was to assess in greater detail the cognitive and sensory-motor behaviour of the MPS II murine model and its progression from 2 to 8 months of age. We demonstrated that MPS II mice exhibit age-dependent behavioural changes in both cognitive and motor function. Many MPS II patients exhibit behavioural difficulties, such as hyperactivity and aggression, and changes in learning abilities have also been described [4]. To this effect, we wanted to investigate whether these traits were also present in the MPS II mouse model at specific ages. The open field apparatus is a well-established paradigm for assessing locomotor activity and anxiety-related behaviour in mice exposed to a novel environment. Anxiety-related behaviour is determined by time spent in the outer zone, as the tendency to remain close to the walls reflects naturalistic anxiety in mice [34]. Open-field testing revealed changes in habituation behaviour at 8 months for the first 10 minutes of the test, suggesting an emotional response to a novel environment. Similarly, hypoactive behaviours have previously been described in 6-, 9- and 10-months-old MPS II mice [6, 19, 35], although no reduction in activity was detected in younger 4–5 months-old cohorts [20]. Hypoactivity and changes in response to a novel environment in the open-field in older mice may be related to the progression of the disease, particularly due to more severe skeletal changes and impaired gait. This finding also highlights the need for longitudinal assessments in behavioural studies. Importantly, no hyperactive behaviour, as is common in MPS II and MPS III patients, was detected in any of our younger cohorts in any phases of the 60-minute test.

In the open field apparatus mice tend to display thigmotaxis by remaining close to the walls, when this pattern changes, either by increasing in the entries or time spent in the centre zone, is thought to reflect a reduction in anxiety-like behaviour. 8-months-old MPS II mice showed an anxiolytic phenotype in the final 20 minutes of the open-field test, spending more time in the centre of the open-field arena. These findings contradict previous studies, which detected anxiety-related behaviour in 6-months and 9-months-old mice in similarly-sized or larger arenas [6, 19]. However, we did not detect any changes in anxiety using the elevated-plus maze in the 8-months-old cohorts. The ten-minute elevated-plus maze test could also be too short to detect any anxiety-related behaviour in MPS II mice, which was only detected after 40 minutes in the open-field arena.

Altered spontaneous alternation is an instrumental feature of cognitive impairment, where short-term storage and retrieval of previous trial choices assesses a particular aspect of spatial working memory [31]. Working memory refers to an aspect of cognition involving temporary storage of information for language, learning and reasoning. In this task, mice have a natural tendency to explore novel environments. This translates into mice preferentially exploring novel arms compared to recently explored arms of the maze [36, 37]. This task can also be used as a proxy measure of overall activity using the number of total arm entries. Importantly, our study shows that the 8-months-old MPS II cohort had impaired spontaneous alternation in the Y-maze with no detectable differences in the number of entries into the arms, suggesting a significant cognitive phenotype in older mice. These findings are consistent with the findings of Wakabayashi and colleagues; however their group size was limited. More importantly, this correlates with the concept that spatial working memory in MPS II mice is impaired at 8 months of age, but not at 9 weeks or 4–5 months of age [18, 20]. Similar findings of decreased spontaneous alternation were detected using the same test in a different MPS II mouse model in 32-week-old mice [38]. The 8-months-old MPS II cohort had normal locomotor activity in the Y-maze.

Psychosocial problems in MPS II are some of the principal problems that parents encounter and pose some of the biggest challenges to families [39, 40]. Aggression, exuberance and over-excitement are commonly cited symptoms. Deficits in social interaction are important markers in developmental disorders. In the present study, a social novelty preference assay was used to investigate sociability and social novel preference in 8-months-old mice only. The specific aim was to establish whether MPS II mice modelled these psychosocial symptoms seen in MPS II patients, and whether these parameters could be used for therapeutic validation. In the sociability phase, MPS II mice spent more time in the chamber containing a stranger mouse over the empty chamber, suggesting that sociability in the MPS II mice remains intact up to at least 8 months of age and is likely independent of other cognitive deficits seen in this mouse model. In the subsequent social novelty preference phase where a second stranger mouse is added, MPS II mice spent more time in the chamber containing the ‘novel’ mouse over the chamber containing the familiar one. Therefore, social interactions in MPS II mice are indistinguishable from WT mice interactions. Recognition of the familiar mouse and identification of the new mouse as socially novel is dependent on retrieval of social memory of the original encounter; therefore this aspect of cognition is not altered in MPS II mice. Interestingly, 4–5 months-old MPS II mice have also been described as having long-term memory deficits, measured using a novel object recognition task [20], having no preference of a novel object over a familiar one.

This study showed a significant neuromuscular phenotype in MPS II mice using the horizontal bar, although age was not considered a factor. MPS II mice generally performed worse than unaffected controls despite large variability between individual mice. In the wire hanging test, no significant differences were observed between WT and MPS II mice at specific ages. However, older cohorts performed significantly worse on the wire hanging test than younger cohorts, suggesting that performance is correlated to age.

MPS II mice also displayed impaired performance on the rotarod at 4 months of age, a well-established test for sensorimotor coordination and balance in models of movement disorders [41, 42]. However, we found no significant differences in 6-month-old and 8-month-old cohorts used in the time course due to variability in our unaffected controls, mainly due to weight gain [43]. This experiment was repeated in independent cohorts of 8-months-old WT and MPS II mice, and MPS II mice showed impairment in coordination and balance. The regulation of coordination, balance and gait in mouse models is a complex interplay between the spinal cord, brain stem, cerebellum, thalamus, basal ganglia, cerebral cortex and muscles [30]. Abnormalities in motor function on the rotarod were similarly detected in a previous study in 10-months-old animals, suggesting that impaired sensorimotor behaviour is detectable early in the MPS II mouse model up to at least 10 months of age and can be used for therapeutic validation [35], provided that body weight in WT control groups is tightly controlled or regulated.

Motor control and balance are regulated by the cerebellum, although it remains unknown whether cerebellar pathology is the sole cause of impaired motor function in the MPS II mice. Vacuolization in Purkinje cells is present at 4 months and 60 weeks of age in MPS II mice [6, 29] and MPS II patients exhibit similar morphological lesions in Purkinje cells [44]. Oxidative markers are also significant altered in the cerebellum of closely-related MPS I mice [45]. Taken together, these data present the notion that cerebellar lesions could have an impact on motor function in MPS II mice. Gait changes, although not investigated in this study, were also identified in 9-months-old MPS II mice [6] but it remains unclear whether this has a direct impact on rotarod performance, as it has been argued that they measure deficits in separate circuits [30].

We also report thickening of zygomatic arches from 2 months of age onwards and of femurs from 4 months of age in MPS II mice, associated with a shortened cranium at 8 months of age and altered facies. Cumulative data from this study and previous work show that the MPS II mouse model accurately recapitulates the severe skeletal phenotype seen in MPS II patients, known as dysostosis multiplex, characterised by restricted mobility, loss of joint range of motion, slow growth and short stature [6, 29, 46].

We have demonstrated that we can detect cognitive and motor differences at 8 months of age in MPS II mice. Older MPS II mice demonstrate altered locomotor behaviour that is likely caused by skeletal changes, and anxiolytic phenotype in extended habituation to a novel environment. This is consistent with the progressive phenotype seen in MPS II patients. Additionally, the MPS II mouse model reliably recapitulates the skeletal abnormalities that are typical of MPS II. The assessment of novel therapies is becoming increasingly reliant on the successful correction of behavioural defects in various animal models, and this study establishes sensitive and reliable tests and time points to obtain robust data in the MPS II mouse model.

## Supporting information

S1 Table Exploratory and anxiety-related behaviour in the open-field test. Distance moved, percentage of time spent in the centre and frequency of entry into the centre were measured over 60 minutes in independent cohorts of WT and MPSII mice (2 months, WT n = 10, MPS II n = 10; 4 months, WT n = 9, MPS II n = 9; 6 months, WT n = 10, MPS II n = 10; 8 months, WT n = 10, MPS II n = 9). Data are expressed as means ± SEM.
(DOCX)

S2 Table Anxiety-related behaviour in the open-field test split into 10-minute time bins. Time spent in the centre of the open-field arena was measured in 10-minute time bins in independent cohorts of WT and MPS II mice (2 months, WT n = 10, MPS II n = 10; 4 months, WT n = 9, MPS II n = 9; 6 months, WT n = 10, MPS II n = 10; 8 months, WT n = 10, MPS II n = 9). Data are expressed as means ± SEM.
(DOCX)

S3 Table Exploratory behaviour in the open-field test split into 10-minute time bins. The distance moved in the centre of the open-field arena was measured in 10-minute time bins in independent cohorts of WT and MPS II mice (2 months, WT n = 10, MPS II n = 10; 4 months, WT n = 9, MPS II n = 9; 6 months, WT n = 10, MPS II n = 10; 8 months, WT n = 10, MPS II n = 9). Data are expressed as means ± SEM.
(DOCX)

S4 Table Spatial working memory testing in the Y-maze in WT and MPS II mice at various ages. Spontaneous alternation and total number of entries were recorded over 10 minutes (2 months, WT n = 10, MPS II n = 10; 4 months, WT n = 9, MPS II n = 7; 6 months, WT n = 10, MPS II n = 10; 8 months, WT n = 9, MPS II n = 8). Data are expressed as means ± SEM.
(DOCX)

S5 Table Neuromuscular phenotype in the inverted screen and on the horizontal bar. Time suspended and the number of hind leg moves were measured on the inverted screen, and the bar crossing score was determined from the horizontal bar test (2 months, WT n = 10, MPS II n = 10; 4 months, WT n = 11, MPS II n = 9; 6 months, WT n = 10, MPS II n = 10; 8 months, WT n = 7, MPS II n = 9). Data are expressed as means ± SEM.
(DOCX)

S6 Table Motor function on the rotarod in WT and MPS II mice. Percentage of time spent on the accelerating rotarod was recorded as an average of three trials at various ages (2 months, WT n = 10, MPS II n = 10; 4 months, WT n = 8, MPS II n = 7; 6 months, WT n = 8, MPS II n = 8; 8 months, WT n = 7, MPS II n = 10). The percentage of time spent on the accelerating rotarod was also measured in a separate 8-months cohort (WT n = 16, MPS II n = 12). Data are expressed as means ± SEM.
(DOCX)

S7 Table Skeletal abnormalities in the MPS II mouse model. Cranial length, zygomatic arch widths and femur widths were measured in independent cohorts of WT and MPS II mice (2 months, WT n = 8, MPS II n = 7; 4 months, WT n = 6, MPS II n = 5; 6 months, WT n = 4, MPS II n = 6; 8 months, WT n = 6, MPS II n = 6. Data are expressed as means ± SEM.
(DOCX)

S8 Table Anxiety-related behaviour in the elevated-plus maze. Distance travelled, percentage of time spent in each zone and the number of entry into each zone were measured in 8-months-old independent cohorts of WT and MPS II mice (WT n = 16, MPS II n = 12). Data are expressed as means ± SEM.
(DOCX)

S9 Table Sociability and social novelty preference in the MPS II mouse model. Sociability and social novelty preference were measured by the percentage of time spent in each chamber over 10-minute periods (WT n = 16, MPS II n = 12). Data are expressed as means ± SEM.
(DOCX)
