# Supplementary material for: Identification of age-dependent motor and neuropsychological behavioural abnormalities in a mouse model of Mucopolysaccharidosis Type II
Source: PLoS One. 2017 Feb 16;12(2):e0172435. doi: 10.1371/journal.pone.0172435 (PMC5313159; doi:10.1371/journal.pone.0172435)
Supplement: S1 Table — Distance moved, percentage of time spent in the centre and frequency of entry into the centre were measured over 60 minutes in independent cohorts of WT and MPSII mice (2 months, WT n = 10, MPS II n = 10; 4 months, WT n = 9, MPS II n = 9; 6 months, WT n = 10, MPS II n = 10; 8 months, WT n = 10, MPS II n = 9). Data are expressed as means ± SEM. (DOCX) [file pone.0172435.s001.docx]

| **Open-field behaviour** | **WT** | | | | **MPS II** | | | |
| --- | --- | --- | --- | --- | --- | --- | --- | --- |
|  | 2 months | 4 months | 6 months | 8 months | 2 months | 4 months | 6 months | 8 months |
| Distance travelled over 60 minutes (cm) | 16412 ±1637.9 | 16120.94  ± 893.9 | 13540.29  ± 766.4 | 16331.14  ± 819.9 | 14039.94 ± 392.1 | 16302.88  ± 850.2 | 14574.06  ± 1040.1 | 14205.44  ± 941.2 |
| Percentage of time spent in centre over 60 minutes | 18.6  ± 2.7 | 19.7  ± 3.1 | 15 ± 2.2 | 16.6 ±  2.1 | 16.46  ±1.7 | 16.6 ± 1.9 | 18.8±2.1 | 23.4±2.9 |
| Frequency of entry into centre zone over 60 minutes | 189.6  ± 22.3 | 199.4 ± 19.2 | 143.4 ± 14.3 | 177.4  ±12.3 | 156.9  ± 13.7 | 180.1  ±15.6 | 174.1  ±14.6 | 186.4  ± 26.3 |

**Table 1. Exploratory and anxiety-related behaviour in the open-field test.** Distance moved, percentage of time spent in the centre and frequency of entry into the centre were measured over 60 minutes in independent cohorts of WT and MPSII mice (2 months, WT n=10, MPS II n=10; 4 months, WT n=9, MPS II n=9; 6 months, WT n=10, MPS II n=10; 8 months, WT n=10, MPS II n=9). Data are expressed as means ± SEM.
