# Supplementary material for: Identification of age-dependent motor and neuropsychological behavioural abnormalities in a mouse model of Mucopolysaccharidosis Type II
Source: PLoS One. 2017 Feb 16;12(2):e0172435. doi: 10.1371/journal.pone.0172435 (PMC5313159; doi:10.1371/journal.pone.0172435)
Supplement: S2 Table — Time spent in the centre of the open-field arena was measured in 10-minute time bins in independent cohorts of WT and MPS II mice (2 months, WT n = 10, MPS II n = 10; 4 months, WT n = 9, MPS II n = 9; 6 months, WT n = 10, MPS II n = 10; 8 months, WT n = 10, MPS II n = 9). Data are expressed as means ± SEM. (DOCX) [file pone.0172435.s002.docx]

| **Open-field behaviour** | | **WT** | | | | **MPS II** | | | |
| --- | --- | --- | --- | --- | --- | --- | --- | --- | --- |
|  |  | 2 months | 4 months | 6 months | 8 months | 2 months | 4 months | 6 months | 8 months |
| Time spent in the centre of the open-field (s) | 0:10:00 | 115.6 ± 18.9 | 112.6 ± 9.1 | 118.1 ± 10.2 | 125.9 ± 10.9 | 134.0 ± 8.2 | 121.0 ± 10.4 | 123.6 ± 21.0 | 96.4 ± 18.9 |
|  | 0:20:00 | 134.3 ± 22.4 | 122.4 ± 17.8 | 127.7 ± 13.1 | 122.8 ± 16.2 | 122.5 ± 14.9 | 102.0 ± 13.0 | 159.2 ± 26.6 | 148.7 ± 25.8 |
|  | 0:30:00 | 102.7 ± 14.8 | 110.8 ± 16.5 | 98.1 ± 19.9 | 111.8 ± 18.1 | 97.6 ± 15.8 | 104.8 ± 13.1 | 132.7 ± 22.8 | 132.4 ± 20.9 |
|  | 0:40:00 | 117.4 ± 18.9 | 115.2 ± 21.2 | 63.6 ± 15.3 | 108.7 ± 21.7 | 76.6 ± 11.1 | 86.4 ± 10.0 | 99.9 ± 17.0 | 132.0 ± 25.7 |
|  | 0:50:00 | 102.6 ± 21.9 | 105.8 ± 19.8 | 72.1 ± 20.2 | 71.4 ± 13.4 | 75.7 ± 13.8 | 107.4 ± 23.2 | 76.8 ± 12.7 | 150.1 ± 17.2 |
|  | 1:00:00 | 97.6 ± 18.9 | 98.6 ± 25.5 | 56.5 ± 16.6 | 58.4 ± 12.5 | 86.4 ± 20.0 | 85.6 ± 15.3 | 83.0 ± 15.2 | 143.3 ± 18.6 |

**Table 2. Anxiety-related behaviour in the open-field test split into 10-minute time bins.** Time spent in the centre of the open-field arena was measured in 10-minute time bins in independent cohorts of WT and MPS II mice (2 months, WT n=10, MPS II n=10; 4 months, WT n=9, MPS II n=9; 6 months, WT n=10, MPS II n=10; 8 months, WT n=10, MPS II n=9). Data are expressed as means ± SEM.
