# Supplementary material for: Identification of age-dependent motor and neuropsychological behavioural abnormalities in a mouse model of Mucopolysaccharidosis Type II
Source: PLoS One. 2017 Feb 16;12(2):e0172435. doi: 10.1371/journal.pone.0172435 (PMC5313159; doi:10.1371/journal.pone.0172435)
Supplement: S3 Table — The distance moved in the centre of the open-field arena was measured in 10-minute time bins in independent cohorts of WT and MPS II mice (2 months, WT n = 10, MPS II n = 10; 4 months, WT n = 9, MPS II n = 9; 6 months, WT n = 10, MPS II n = 10; 8 months, WT n = 10, MPS II n = 9). Data are expressed as means ± SEM. (DOCX) [file pone.0172435.s003.docx]

| **Open-field behaviour** | | **WT** | | | | **MPS II** | | | |
| --- | --- | --- | --- | --- | --- | --- | --- | --- | --- |
|  |  | 2 months | 4 months | 6 months | 8 months | 2 months | 4 months | 6 months | 8 months |
| Distance moved in the open-field per 10min time bin (cm) | 0:10:00 | 3538.1 ± 263.8 | 3359.6 ± 256.2 | 2940.5 ± 125.3 | 3592.4 ± 225.3 | 3368.7 ± 178.7 | 3051.8 ± 133.3 | 2632.6 ± 193.3 | 2758.1 ± 178.9 |
|  | 0:20:00 | 2989.5 ± 301.5 | 2736.6 ± 211.7 | 2612.4 ± 175.0 | 3007.4 ± 145.2 | 2587.7 ± 109.5 | 2788.9 ± 169.2 | 2838.1 ± 153.7 | 2434.1 ± 175.8 |
|  | 0:30:00 | 2623.2± 252.8 | 2528.5 ± 166.2 | 2513.9 ± 178.9 | 2929.5 ± 131.3 | 2297.9 ± 113.9 | 2970.1 ± 176.9 | 2585.0 ± 245.3 | 2284.7 ± 182.0 |
|  | 0:40:00 | 2479.4 ± 260.3 | 2469.4 ± 161.3 | 2067.5 ± 185.0 | 2542.1 ± 152.1 | 2062.2 ± 89.4 | 2554.1 ± 174.5 | 2347.6 ± 209.9 | 2169.4 ± 166.7 |
|  | 0:50:00 | 2440.7± 340.7 | 2212.3 ± 169.7 | 1732.2 ± 165.8 | 2229.2 ± 174.3 | 1936.7 ± 120.1 | 2558.3 ± 96.1 | 2220.6 ± 233.7 | 2431.5 ± 158.2 |
|  | 1:00:00 | 2341.1 ± 339.7 | 1982.4 ± 141.6 | 1673.7 ± 180.7 | 2030.6 ± 168.4 | 1786.8 ± 86.2 | 2311.3 ± 195.6 | 1950.3 ± 154.7 | 2185.0 ± 145.9 |

**Table 3. Exploratory behaviour in the open-field test split into 10-minute time bins.** The distance moved in the centre of the open-field arena was measured in 10-minute time bins in independent cohorts of WT and MPS II mice (2 months, WT n=10, MPS II n=10; 4 months, WT n=9, MPS II n=9; 6 months, WT n=10, MPS II n=10; 8 months, WT n=10, MPS II n=9). Data are expressed as means ± SEM.
