# Supplementary material for: Identification of age-dependent motor and neuropsychological behavioural abnormalities in a mouse model of Mucopolysaccharidosis Type II
Source: PLoS One. 2017 Feb 16;12(2):e0172435. doi: 10.1371/journal.pone.0172435 (PMC5313159; doi:10.1371/journal.pone.0172435)
Supplement: S4 Table — Spontaneous alternation and total number of entries were recorded over 10 minutes (2 months, WT n = 10, MPS II n = 10; 4 months, WT n = 9, MPS II n = 7; 6 months, WT n = 10, MPS II n = 10; 8 months, WT n = 9, MPS II n = 8). Data are expressed as means ± SEM. (DOCX) [file pone.0172435.s004.docx]

| **Y-maze behaviour** | **WT** | | | | **MPS II** | | | |
| --- | --- | --- | --- | --- | --- | --- | --- | --- |
|  | 2 months | 4 months | 6 months | 8 months | 2 months | 4 months | 6 months | 8 months |
| Percentage alternation | 63.07  ± 3.6 | 65.39 ± 4.4 | 61.1  ± 2.8 | 61.7  ± 0.9 | 64.0  ± 2.4 | 64.7  ± 5.4 | 66.2  ± 3.1 | 55.2  ± 2.3 |
| Total number of entries | 38 ± 3.1 | 33.5 ± 3.5 | 27.4± 2.9 | 29 ± 2.6 | 28.1 ± 2.8 | 31.2 ±  3.3 | 24 ± 1.4 | 31.1 ± 3.4 |

**Table 4. Spatial working memory testing in the Y-maze in WT and MPS II mice at various ages.** Spontaneous alternation and total number of entries were recorded over 10 minutes (2 months, WT n=10, MPS II n=10; 4 months, WT n=9, MPS II n=7; 6 months, WT n=10, MPS II n=10; 8 months, WT n=9, MPS II n=8). Data are expressed as means ± SEM.
