# Supplementary material for: Identification of age-dependent motor and neuropsychological behavioural abnormalities in a mouse model of Mucopolysaccharidosis Type II
Source: PLoS One. 2017 Feb 16;12(2):e0172435. doi: 10.1371/journal.pone.0172435 (PMC5313159; doi:10.1371/journal.pone.0172435)
Supplement: S5 Table — Time suspended and the number of hind leg moves were measured on the inverted screen, and the bar crossing score was determined from the horizontal bar test (2 months, WT n = 10, MPS II n = 10; 4 months, WT n = 11, MPS II n = 9; 6 months, WT n = 10, MPS II n = 10; 8 months, WT n = 7, MPS II n = 9). Data are expressed as means ± SEM. (DOCX) [file pone.0172435.s005.docx]

| **Neuromuscular phenotype** | **WT** | | | | **MPS II** | | | |
| --- | --- | --- | --- | --- | --- | --- | --- | --- |
|  | 2 months | 4 months | 6 months | 8 months | 2 months | 4 months | 6 months | 8 months |
| Suspended time on inverted screen (s) | 118 ± 2 | 98.3 ±10.7 | 85.6  ±  10.2 | 80.4  ±  10.6 | 104.2  ±  6.9 | 88.4  ±  10.0 | 90.1  ±  12.1 | 74.3  ±  8.9 |
| Hind leg moves on inverted screen | 52.5  ±  7.0 | 51.1  ±  6.7 | 43.7  ±  8.1 | 44.6 ±  7.1 | 56.5  ±  5.7 | 44.8 ±  7.2 | 46.7  ±  6.8 | 32.6 ±  5.3 |
| Bar crossing score | 228.1  ±  1.2 | 228.37  ±  1.8 | 213.4  ±  9.8 | 212.1  ±  13.7 | 228.6  ±  2.9 | 169.6  ±  30.3 | 182.1  ±  21.8 | 177.2  ±  27.6 |

**Table 5. Neuromuscular phenotype in the inverted screen and on the horizontal bar**. Time suspended and the number of hind leg moves were measured on the inverted screen, and the bar crossing score was determined from the horizontal bar test (2 months, WT n=10, MPS II n=10; 4 months, WT n=11, MPS II n=9; 6 months, WT n=10, MPS II n=10; 8 months, WT n=7, MPS II n=9). Data are expressed as means ± SEM.
