# Supplementary material for: Identification of age-dependent motor and neuropsychological behavioural abnormalities in a mouse model of Mucopolysaccharidosis Type II
Source: PLoS One. 2017 Feb 16;12(2):e0172435. doi: 10.1371/journal.pone.0172435 (PMC5313159; doi:10.1371/journal.pone.0172435)
Supplement: S6 Table — Percentage of time spent on the accelerating rotarod was recorded as an average of three trials at various ages (2 months, WT n = 10, MPS II n = 10; 4 months, WT n = 8, MPS II n = 7; 6 months, WT n = 8, MPS II n = 8; 8 months, WT n = 7, MPS II n = 10). The percentage of time spent on the accelerating rotarod was also measured in a separate 8-months cohort (WT n = 16, MPS II n = 12). Data are expressed as means ± SEM. (DOCX) [file pone.0172435.s006.docx]

| **Rotarod** | **WT** | | | | **MPS II** | | | |
| --- | --- | --- | --- | --- | --- | --- | --- | --- |
|  | 2 months | 4 months | 6 months | 8 months | 2 months | 4 months | 6 months | 8 months |
| Percentage time on the rotarod | 73.9 ± 6.1 | 71.7 ± 5.2 | 36.9 ± 2.9 | 62.5 ± 10.4 | 71.4 ± 5.3 | 55.7 ± 4.3 | 51.1 ± 6.6 | 49.4 ± 4.5 |
| Percentage time on the rotarod –  8-months repeat | N/A | N/A | N/A | 57.5 ± 3.5 | N/A | N/A | N/A | 43.3 ± 3.9 |

**Table 6. Motor function on the rotarod in WT and MPS II mice.** Percentage of time spent on the accelerating rotarod was recorded as an average of three trials at various ages (2 months, WT n=10, MPS II n=10; 4 months, WT n=8, MPS II n=7; 6 months, WT n=8, MPS II n=8; 8 months, WT n=7, MPS II n=10). The percentage of time spent on the accelerating rotarod was also measured in a separate 8-months cohort (WT n=16, MPS II n=12). Data are expressed as means ± SEM.
