# Supplementary material for: Identification of age-dependent motor and neuropsychological behavioural abnormalities in a mouse model of Mucopolysaccharidosis Type II
Source: PLoS One. 2017 Feb 16;12(2):e0172435. doi: 10.1371/journal.pone.0172435 (PMC5313159; doi:10.1371/journal.pone.0172435)
Supplement: S7 Table — Cranial length, zygomatic arch widths and femur widths were measured in independent cohorts of WT and MPS II mice (2 months, WT n = 8, MPS II n = 7; 4 months, WT n = 6, MPS II n = 5; 6 months, WT n = 4, MPS II n = 6; 8 months, WT n = 6, MPS II n = 6. Data are expressed as means ± SEM. (DOCX) [file pone.0172435.s007.docx]

| **Skeletal abnormalities** | **WT** | | | | **MPS II** | | | |
| --- | --- | --- | --- | --- | --- | --- | --- | --- |
|  | 2 months | 4 months | 6 months | 8 months | 2 months | 4 months | 6 months | 8 months |
| Cranial length (mm) | 22.056 ±  0.302 | 22.560 ±  0.334 | 23.326 ±  0.103 | 22.935 ±  0.430 | 22.199 ±  0.222 | 21.943 ±  0.508 | 21.427 ±  0.267 | 20.987 ±  0.593 |
| Zygomatic arch width (mm) | 0.469 ±  0.012 | 0.469 ±  0.013 | 0.463 ±  0.039 | 0.492 ±  0.023 | 0.648 ±  0.023 | 0.781 ±  0.029 | 0.950 ±  0.020 | 0.939 ±  0.035 |
| Femur width (mm) | 1.427 ±  0.034 | 1.425 ±  0.038 | 1.392 ±  0.049 | 1.457 ±  0.018 | 1.565 ±  0.032 | 1.811 ±  0.062 | 1.781 ±  0.049 | 1.842 ±  0.060 |

**Table 7. Skeletal abnormalities in the MPS II mouse model**. Cranial length, zygomatic arch widths and femur widths were measured in independent cohorts of WT and MPS II mice (2 months, WT n=8, MPS II n=7; 4 months, WT n=6, MPS II n=5; 6 months, WT n=4, MPS II n=6; 8 months, WT n=6, MPS II n=6. Data are expressed as means ± SEM.
