# Supplementary material for: Identification of age-dependent motor and neuropsychological behavioural abnormalities in a mouse model of Mucopolysaccharidosis Type II
Source: PLoS One. 2017 Feb 16;12(2):e0172435. doi: 10.1371/journal.pone.0172435 (PMC5313159; doi:10.1371/journal.pone.0172435)
Supplement: S8 Table — Distance travelled, percentage of time spent in each zone and the number of entry into each zone were measured in 8-months-old independent cohorts of WT and MPS II mice (WT n = 16, MPS II n = 12). Data are expressed as means ± SEM. (DOCX) [file pone.0172435.s008.docx]

| **Elevated-plus maze** | **WT** | | **MPS II** | |
| --- | --- | --- | --- | --- |
| Distance travelled (cm) | 1174.8 ± 112.5 | | 895.6 ± 80.7 | |
|  | Closed arm | Open arm | Closed arm | Open arm |
| Percentage of duration in each zone | 47.7 ± 6.4 | 23.6 ± 6.6 | 45.0 ± 3.7 | 26.2 ± 5.1 |
| Number of arm entries | 13.4 ± 2.6 | 12.4 ± 2.0 | 9.0 ± 1.0 | 13.2 ± 1.3 |

**Table 8. Anxiety-related behaviour in the elevated-plus maze.** Distance travelled, percentage of time spent in each zone and the number of entry into each zone were measured in 8-months-old independent cohorts of WT and MPS II mice (WT n=16, MPS II n=12). Data are expressed as means ± SEM.
