# Supplementary material for: Identification of age-dependent motor and neuropsychological behavioural abnormalities in a mouse model of Mucopolysaccharidosis Type II
Source: PLoS One. 2017 Feb 16;12(2):e0172435. doi: 10.1371/journal.pone.0172435 (PMC5313159; doi:10.1371/journal.pone.0172435)
Supplement: S9 Table — Sociability and social novelty preference were measured by the percentage of time spent in each chamber over 10-minute periods (WT n = 16, MPS II n = 12). Data are expressed as means ± SEM. (DOCX) [file pone.0172435.s009.docx]

| **Sociability & social preference** | **WT** | | **MPS II** | |
| --- | --- | --- | --- | --- |
| **Sociability** | **Empty chamber** | **Stranger mouse** | **Empty chamber** | **Stranger mouse** |
| Percentage duration in zone | 26.3 ± 1.5 | 44.7 ± 2.2 | 28.2 ± 2.2 | 46.1 ± 3.4 |
| **Social Preference** | **Familiar mouse** | **Stranger mouse** | **Familiar mouse** | **Stranger mouse** |
| Percentage duration in zone | 30.4 ± 3.4 | 44.5 ± 3.6 | 24.8 ± 2.0 | 43.7 ± 2.7 |

**Table 9. Sociability and social novelty preference in the MPS II mouse model.** Sociability and social novelty preference were measured by the percentage of time spent in each chamber over 10-minute periods (WT n=16, MPS II n=12). Data are expressed as means ± SEM.
